# Characterizing soluble immune checkpoint molecules and TGF-β_1,2,3_ in pleural effusion of malignant pleural mesothelioma

**DOI:** 10.1038/s41598-024-66189-5

**Published:** 2024-07-10

**Authors:** Riki Okita, Tomoya Senoo, Yuka Mimura-Kimura, Yusuke Mimura, Tomoyuki Murakami, Eiji Ikeda, Masanori Okada, Hidetoshi Inokawa, Keisuke Aoe

**Affiliations:** 1 Department of Thoracic Surgery, National Hospital Organization Yamaguchi Ube Medical Center, Higashikiwa 685, Ube, Yamaguchi 755-0241 Japan; 2 Department of Clinical Research, National Hospital Organization Yamaguchi Ube Medical Center, Higashikiwa 685, Ube, Yamaguchi 755-0241 Japan; 3 Department of Pathology, KYURIN/KYURIN PACELL Corporation, 26-67 Morishita-Cho, Yahatanishi-Ku, Kitakyushu, Fukuoka 806-0046 Japan; 4 Department of Pathology, Yamaguchi University Graduate School of Medicine, 1-1-1 Minami-Kogushi, Ube, Yamaguchi 755-8505 Japan; 5 Department of Medical Oncology, National Hospital Organization Yamaguchi Ube Medical Center, Higashikiwa 685, Ube, Yamaguchi 755-0241 Japan

**Keywords:** Mesothelioma, Pleural effusion, Tumor immune microenvironment, Immune checkpoint molecule, TGF-β, Cancer microenvironment, Mesothelioma, Tumour biomarkers, Tumour immunology, Cancer, Oncology

## Abstract

The clinical impact of soluble molecules in pleural effusion (PE) is unclear in patients with malignant pleural mesothelioma (MPM). In this single-center, retrospective, observational study, we assessed soluble forms of cytotoxic T-lymphocyte-associated protein 4 (CTLA-4), programmed cell death protein 1 (PD-1), and PD-1 ligand 1 (PD-L1) using enzyme-linked immunosorbent assays; three TGF-β isoforms were measured via multiplex assay in PE of patients with fibrinous pleuritis (FP) or MPM, to assess relationships between the levels of six molecules, clinicopathological characteristics, and efficacy of immune checkpoint inhibitors. Soluble forms of CTLA-4, PD-L1, PD-1, TGF-β_1_, TGF-β_2_, and TGF-β_3_ were variably produced in PE of FP (n = 34) and MPM (n = 79); we found significant relationships between the six molecules and clinicopathological features. Although none of the three soluble immune checkpoint molecules showed diagnostic or prognostic effects in patients with MPM, TGF-β_2_ level in PE is a useful differential diagnostic marker between FP and MPM. Both TGF-β_1_ and TGF-β_3_ levels are promising prognostic markers for MPM. Moreover, we found that higher baseline levels of PD-1 soluble forms predicted the response to anti-PD1 monotherapy. Our findings identify novel diagnostic, prognostic, and predictive biomarkers for anti-PD1 therapy in patients with MPM.

## Introduction

Malignant pleural mesothelioma (MPM) is an aggressive solid tumor whose onset is strongly associated with asbestos exposure-induced chronic inflammation^1^. Recently, immune checkpoint inhibitors (ICIs) targeting programmed cell death protein 1 (PD-1)/PD-1 ligand-1 (PD-L1) axis and cytotoxic T-lymphocyte-associated protein 4 (CTLA-4) have shown improved clinical outcomes in patients with MPM^2^, indicating that immune escape from host immunity is a primary mechanism of MPM tumor progression. Clinical studies revealed that PD-L1-overexpressed tumors showed better clinical responses compared to PD-L1 low-expressing tumors in several malignancy types^3,4^, suggesting that PD-L1 overexpression in tumor tissues is a potential biomarker for ICI targeting PD-1/PD-L1; however, an objective response was seen in both PD-L1 negative and positive MPM^2^, implying that the MPM tumor immune escape mechanism is complicated. Other than PD-L1 expression, meta-analytical studies have explored predictive factors for ICI; notably, the development of immune-related adverse events was associated with a durable response and survival benefit in patients with solid malignancies^5^. Furthermore, pretreatment hematological markers such as a lower neutrophil-to-lymphocyte ratio or higher albumin levels also predicted a better clinical outcome^6,7^. Importantly, clinical benefits were similarly observed between cases with Eastern Cooperative Oncology Group performance status 0 and 1^8^. Currently, none of these factors can be used in the decision-making process with ICI.

The tumor immune microenvironment (TIME), consisting of tumor, immune, vascular, and stromal cells, is important for facilitating the tumor cell escape from anti-tumor immunity via immune suppressive molecules such as immune checkpoint molecules (ICMs) and immune suppressive cytokines^9^. The TIME is an important topic for cancer research because other than the PD-L1 status, TIME can predict ICI efficacy in melanoma^10^. Although studying TIME of MPM is difficult because of its rarity, several studies have shown that PD-L1 overexpression in tumor tissue using immunohistochemical staining (IHC) predicts poor prognosis^11,12^, whereas many other studies could not show any impact of PD-L1 status on the prognosis of patients with MPM^13,14^.

In many cases, MPM is accompanied by pleural effusion (PE), which could also be considered as a TIME. To date, only a single assessment of the soluble form of CTLA-4 (sCTLA-4) or PD-L1 (sPD-L1) has been conducted in the PE of MPM^15,16^, and the simultaneous evaluation of multiplex soluble ICMs has not been reported. Transforming growth factor (TGF)-β is rich in MPM^17,18^, and drives epithelial-mesenchymal transition (EMT) and inactivates anti-cancer immunity, making its involvement in PE worth investigating^19,20^. TGF-β has three isoforms; TGF-β_1_, TGF-β_2,_ and TGF-β_3_^21^. Only one report has evaluated TGF-β levels in PE, wherein more than 10 cases of MPM were investigated^18^; however, levels of the three TGF-β isoforms in PE of MPM have not been reported.

Here, we first evaluate concentrations of multiplex soluble ICMs: sCTLA-4, sPD-L1, and PD-1 soluble form (sPD-1), and three TGF-β isoforms in PE of fibrinous pleuritis (FP) or MPM. TGF-β_2_ was higher in PE of MPM than in PE of FP, suggesting that TGF-β_2_ levels in PE could be a useful tool for differential diagnosis between MPM and FP. The three soluble ICMs did not show any prognostic impact, whereas higher concentrations of both TGF-β_1_ and TGF-β_3_ predicted poor overall survival (OS) in patients with MPM. Moreover, we found that a higher baseline level of sPD-1 in PE predicted the response to anti-PD-1 monotherapy in patients with MPM.

## Materials and methods

### Patients

This was a single-center, retrospective, observational study. PE samples were collected for cytology and/or biochemistry tests from patients with FP or MPM prior to treatment, except for three patients with postoperative recurrence for whom extrapleural pneumonectomy with or without perioperative chemotherapy was performed, between February 2007 and July 2022. The residual PE samples were centrifuged at 800 × g at 4 °C for 10 min and the supernatants were frozen at − 80 °C until analysis. All cases were histologically diagnosed as FP or MPM by experienced pathologists using IHC. The 5-year OS was estimated from the date of PE collection to death from any cause or the last follow-up. The requirement of written informed consent for using medical records, tissue samples, and PE samples was waived, and the opt-out method on the website was used to obtain patient consent. This observational study adhered to the principles of the Declaration of Helsinki and complied with relevant guidelines and regulations, and it was approved by the Yamaguchi Ube Medical Center Ethics Committee (No. 28-13, 30-5, and 2020-17).

### Clinical variables

The following patient characteristics were obtained from medical records for analysis: age, sex, histological subtype, clinical stage (cStage), and treatment (surgery, radiotherapy, chemotherapy, and ICIs). The follow-up period was set at a maximum of five years (60 months). Histological subtype and cStage were determined according to the criteria from the World Health Organization 2015^22^ and the eighth edition of the TNM stage classification system^23–25^, respectively. The last follow-up date was August 31, 2023, and the median length of follow-up for censored cases was 13.4 months (range, 4.7–33.4 months).

### BAP1 status and tumor infiltrating CD8 T cell (TIL) using IHC

Formalin-fixed and paraffin-embedded blocks of MPM were obtained from the institution archives. IHC for BRCA1 associated protein 1 (BAP1) was performed on surgically removed tissues using an automated immunostainer (Ventana BenchMark GX; Roche Diagnostics, Basel, Switzerland). Briefly, slides were deparaffinized using the EZ buffer (Roche Diagnostics) for 1 min at 75 °C and antigen was retrieved using Cell Conditioning 1 buffer (Roche Diagnostics) at 100 °C for 60 min. In addition, a mouse monoclonal anti-BAP1 antibody (1:50, clone C-4, Santa Cruz Biotechnology, Dallas, TX) was used as the primary antibody at 37 °C for 32 min, followed by secondary antibody and detection using the Ventana UltraView Universal DAB detection kit (Roche Diagnostics) as per manufacturer’s instructions. Loss of BAP1 was defined as the absence of BAP1 staining in the MPM cell nuclei. Immunoreactivity for BAP1 (loss or retention) was assessed by experienced pathologists (T.M. or E.I.). Nucleus staining for BAP1 in lymphocytes and vascular endothelial cells was used as an internal positive control for BAP1 expression. IHC for CD8 TIL was performed as double staining with rabbit anti-CD8α IgG antibody (clone D8A8Y, Cell Signaling Technology, Danvers, MA) and mouse anti-EGFR IgG2a antibody (clone A-10, Santa Cruz Biotechnology); the status of CD8 TIL was judged by two scorers (R.O. and T.S.) following previously described protocol^14^.

### ELISA

The levels of sCTLA-4, sPD-L1, and sPD-1 in PE were measured using enzyme-linked immunosorbent assays (ELISA) using a Human CTLA-4 Quantikine HS ELISA kit (#HSCT40, R&D Systems, Minneapolis, MN), Human/Cynomolgus Monkey B7-H1/PD-L1 Quantikine ELISA kit (#DB7H10, R&D Systems), and Human PD-1 Quantikine HS ELISA kit (#DPD10, R&D Systems), respectively, as per manufacturer’s instructions. The minimum detectable doses of sCTLA-4, sPD-L1, and sPD-1 were 0.13, 4.52, and 3.27 pg/ml, respectively. The plates were read at 450 nm using a Multiskan FC (Thermo Fisher Scientific, Waltham, MA).

### Multiplex cytokine detecting assay

TGF-β_1,2,3_ levels in PE were measured via multiplex assay using Bio-Plex Pro Human TGF-β 3-plex panel (#171W4001M, Bio-Rad, Hercules, CA), as per manufacturer’s instructions. The minimum detectable doses of TGF-β_1_, TGF-β_2_, and TGF-β_3_ were 3.9, 1.9, and 0.5 pg/ml, respectively. Plates were read using a Bio-Plex 200 system (Bio-Rad).

### Statistical analysis

The cutoff values of each parameter were individually determined according to the receiver operating characteristic (ROC) curves for differential diagnosis between FP and MPM or for predicting 2-year survival or response to ICI in cases of MPM. A *t*-test was performed to compare the levels of the six PE parameters between the FP and MPM groups or the historical subtypes of MPM. A chi-square test was performed to evaluate the relationship between patient characteristics and TIME parameters. Spearman’s rank correlation test was performed for correlation analysis between the levels of the six PE parameters. Kaplan–Meier survival analysis was used to determine the association between the status of each parameter and the 5-year OS until death or last follow-up. The significant differences in the 5-year OS between groups were assessed using the log-rank test. Statistical analyses were performed using GraphPad Prism 6.01 (GraphPad Software, La Jolla, CA). Univariate and multivariate analyses were performed with the Cox proportional hazards model to identify independent prognostic factors using the SPSS statistical package (version 17.0; SPSS, Chicago, IL). In all cases, statistical significance was set at *p* < 0.05.

### Ethics approval and consent to participate

This research was approved by the Yamaguchi Ube Medical Center Ethics Committee (No. 28-13, 30-5 and 2020-17), followed the principles of the Declaration of Helsinki, and complied with the relevant guidelines and regulations. The requirement for written informed consent for the use of medical records was waived and an opt-out method on the website was used to obtain patient consent.

## Results

### Patient cohort

Overall, 113 patients were enrolled and our cohort comprised 34 and 79 patients with FP and MPM, respectively. A definitive pathological diagnosis was performed via pleural biopsy in all patients with MPM at our hospital (72) or elsewhere (7). Patient backgrounds are shown in Table 1.

**Table 1:** Clinicopathological characteristics of patients.

Characteristics	FP	MPM
Age: average ± S.D. (n = 79)	68.8 ± 10.0	71.2 ± 9.7
Sex (n = 79)		
Male	33	68
Female	1	11
Histology (n = 79)		
Epithelioid		47
Biphasic		25
Sarcomatoid		7
cStage (8th TNM) (n = 79)		
IA, B		59
II		4
IIIA, B		11
IV or rec		5
BAP1 status (n = 72)		
Loss		46
Retained		26
CD8 TIL status (n = 72)		
Negative		36
Positive		36

*FP* fibrious pleuritic, *MPM* malignant pleural mesothelioma, *S.D.* standard deviation, *rec.* recurrence after surgery, *BAP1* BRCA1 associated protein 1, *CD8 TIL* tumor infiltrating CD8 T cell.

### ***Levels of three soluble ICMs and TGF-β***_***1,2,3***_*** in PE of FP or MPM***

**Figure 1:**
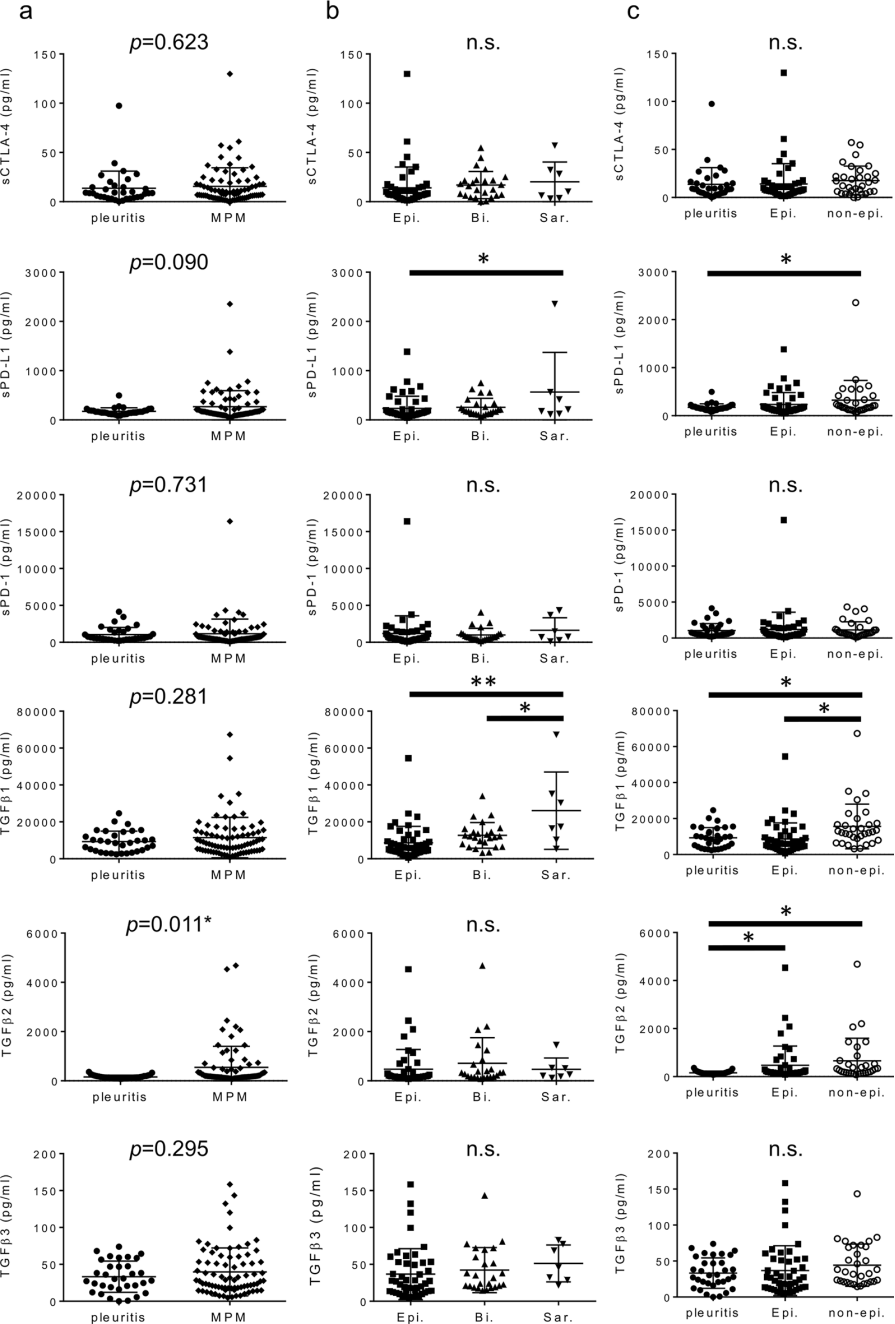
Levels of six parameters in PE of patients with FP or MPM. (**a**) Each dot represents the levels of sCTLA4, sPD-L1, sPD-1, TGF-β_1_, TGF-β_2_, and TGF-β_3_ in the PE of FP or MPM, (**b**) in the PE of epithelioid, biphasic, or sarcomatoid MPM, and (**c**) in the PE of FP, epithelioid, or non-epithelioid (biphasic and sarcomatoid) MPM. Data were analyzed using a *t*-test. Bars: Mean ± SD. *p < 0.05, **p < 0.001. *PE* pleural effusion, *FP* fibrinous pleuritic, *MPM* malignant pleural mesothelioma, *sCTLA-4* soluble CTLA-4, *sPD-L1* soluble PD-L1, *sPD-1* soluble PD1, *Epi.* epithelioid MPM, *Bi.* biphasic MPM, *Sar.* sarcomatoid MPM, *SD* standard deviation, *n.s.* not significant.

The mean (± standard deviation) concentrations of the three soluble ICM (sCLTA-4, sPD-L1, and sPD-1) and three TGF-β (TGF-β_1_, TGF-β_2_, and TGF-β_3_) isoforms in PE of FP were 13.64 ± 17.46, 173.0 ± 74.50, 1040 ± 966.2, 9277 ± 5709, 159.0 ± 69.53, and 33.30 ± 21.16 pg/ml, whereas those in PE of MPM were 15.51 ± 18.91, 269.4 ± 324.5, 1163 ± 1978, 11,465 ± 10,967, 545.8 ± 858.1, and 39.78 ± 32.55 pg/ml, respectively. TGF-β_2_ was significantly higher in PE of MPM than in PE of FP (*p* = 0.011). The sPD-L1 level in PE of MPM tended to be higher than that in PE of FP, although the difference was not significant (*p* = 0.090). No difference was observed between FP and MPM in the other four parameters (Fig. 1a and Supplementary Table S1). Optimal cutoff values for differential diagnosis between FP and MPM were determined via ROC analyses as follows: 9.19 pg/ml for sCTLA-4, 163.46 pg/ml for sPD-L1, 581.15 pg/ml for sPD-1, 8413.45 pg/ml for TGF-β_1_, 177.99 pg/ml for TGF-β_2_, and 28.56 pg/ml for TGF-β_3_. The higher TGF-β_2_ level was a significant diagnostic parameter for MPM (*p* = 0.0004), whereas the other five parameters were not significant (Supplementary Fig. S1).

The six parameters in each MPM histology type were analyzed. The sPD-L1 level in PE of sarcomatoid MPM was significantly higher (565.4 ± 805.6 pg/ml) than that in PE of epithelioid MPM (232.8 ± 247.8 pg/ml, *p* = 0.027) and higher, although not significantly, than that in PE of biphasic MPM (255.4 ± 184.3 pg/ml, *p* = 0.077). TGF-β_1_ in PE of sarcomatoid MPM (26,084 ± 20,975 pg/ml) was higher than that in PE of epithelioid MPM (8649 ± 8929 pg/ml, *p* = 0.0003) or biphasic MPM (12,667 ± 6978 pg/ml, *p* = 0.009) (Fig. 1b). When comparing levels of the six parameters between epithelioid and non-epithelioid (biphasic + sarcomatoid) MPM, significant differences were observed in sPD-L1 levels between FP and non-epithelioid MPM (323.2 ± 410.9 pg/ml, *p* = 0.040), TGF-β_1_ levels between non-epithelioid MPM (15,602 ± 12,434 pg/ml) and FP (*p* = 0.010) or epithelioid MPM (*p* = 0.005), and TGF-β_2_ levels between FP and epithelioid MPM (472.2 ± 802.8 pg/ml, *p* = 0.029) or non-epithelioid MPM (657.4 ± 938.2 pg/ml, *p* = 0.003) (Fig. 1c).

### Correlations between the six PE parameters in patients with MPM

Correlations between each soluble ICM and TGF-β_1,2,3_ were assessed. Significant positive correlations were observed between sCTLA-4 and sPD-L1 [Spearman’s rank correlation coefficient (Rs): 0.207, *p* < 0.0001], sCTLA-4 and sPD-1 (Rs: 0.755, *p* < 0.0001), sPD-L1 and sPD-1 (Rs: 0.125, *p* = 0.001), sPD-L1 and TGF-β_1_ (Rs: 0.053, *p* = 0.040), TGF-β_1_ and TGF-β_3_ (Rs: 0.190, *p* < 0.0001), and TGF-β_2_ and TGF-β_3_ (Rs: 0.193, *p* < 0.0001) (Supplementary Fig. S2) levels. The correlation between sCTLA-4 and sPD-1 was strong, whereas that between sCTLA-4 and sPD-L1 was moderate; correlations between sPD-L1 and sPD-1, sPD-L1 and TGF-β_1_, TGF-β_1_ and TGF-β_3_, and TGF-β_2_ and TGF-β_3_ were weak.

### Relationships between the six PE parameters and BAP1 and CD8 TIL statuses in MPM

Proteomic data from MPM showed that BAP1-deficient tumors were characterized by inflammatory TIME and activation of immune checkpoint mechanisms^26^. Therefore, the relationship between the six PE parameters and the statuses of BAP1 and CD8 TIL in MPM was analyzed. Tissue blocks from 72 patients with MPM were obtained from the institution archives, whereas blocks from the other seven cases could not be assessed because the biopsy was performed outside our hospital. Representative images of BAP1 and CD8 + TILs are shown in Supplementary Figure S3. The BAP1 status was considered a “loss” in 46 cases and “retained” in 26 cases of MPM, whereas the CD8 TILs were negative in 36 cases and positive in 36 cases of MPM. TGF-β_1_ in PE of MPM was significantly lower in cases with BAP1 loss tumors (9218 ± 6776 pg/ml) than in cases with BAP1-retained tumors (15,474 ± 15,944 pg/ml, *p* = 0.023). The sPD-L1 level in PE of MPM was lower in BAP1 loss tumors (208.0 ± 167.4 pg/ml) than in BAP1-retained tumors (338.7 ± 457.4 pg/ml, *p* = 0.085), although the difference was not significant (Supplementary Fig. S4a). The sCTLA-4 level in PE of MPM was lower in cases without CD8 TILs (10.41 ± 8.390 pg/ml) than in those with CD8 TILs (19.70 ± 25.35 pg/ml. *p* = 0.041), whereas TGF-β_1_ in PE of MPM was higher in cases without CD8 TILs (14,105 ± 14,598 pg/ml) than in those with CD8 TILs (8849 ± 5646 pg/ml, *p* = 0.048) (Supplementary Fig. S4b).

### OS stratified by the six PE parameters, BAP1, and CD8 TIL in MPM

**Figure 2:**
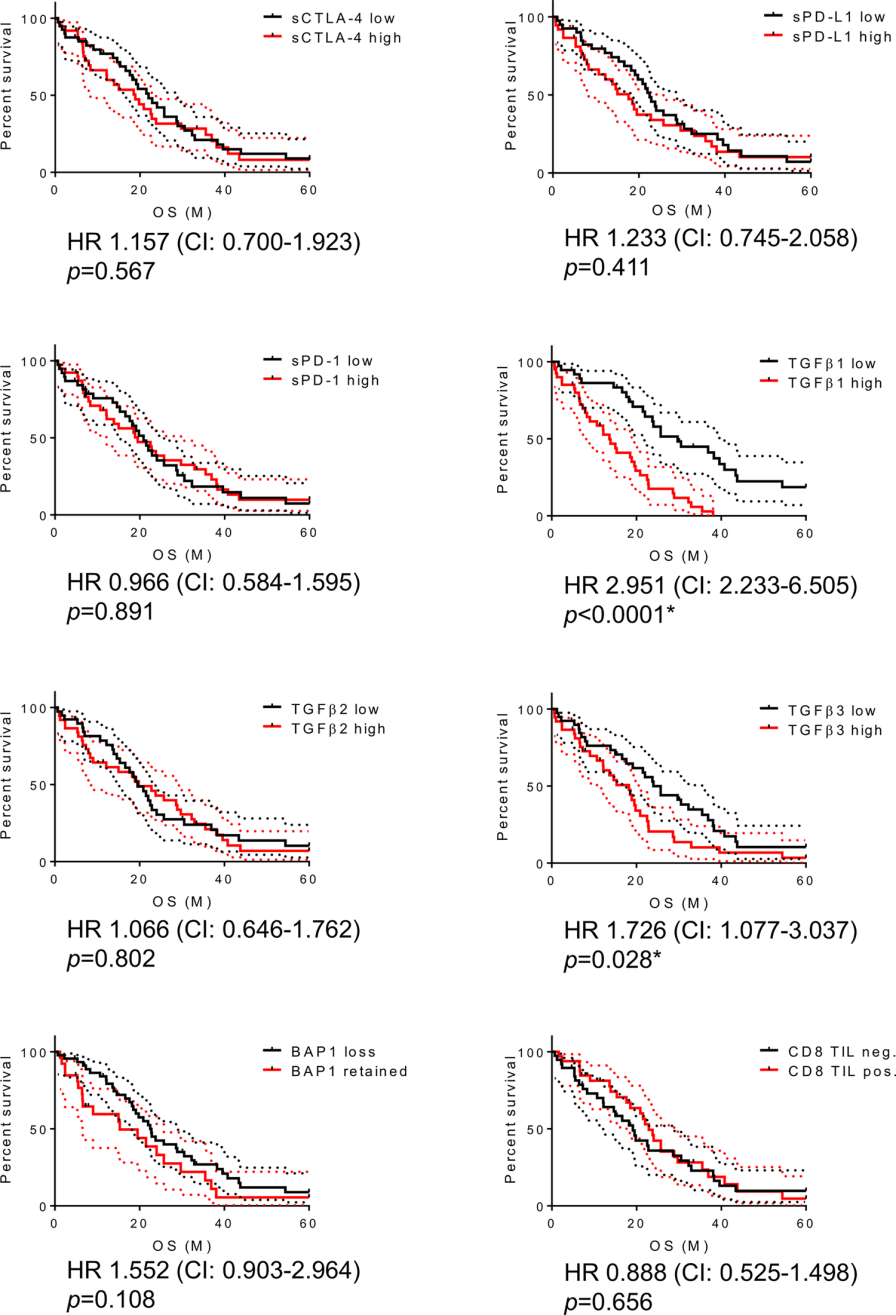
Associations of the six parameters and BAP1 and CD8 TIL statuses in PE with 5-year overall survival in patients with MPM. Kaplan–Meier plots showing 5-year overall survival in patients with low or high sCTLA-4 (cutoff: 9.555 pg/ml), sPD-L1 (cutoff: 165.73 pg/ml), sPD-1 (cutoff: 601.5 pg/ml), TGF-β_1_ (cutoff: 8100.75 pg/ml), TGF-β_2_ (cutoff: 198.275 pg/ml), and TGF-β_3_ (cutoff: 28.555 pg/ml) levels in PE; loss or retention of BAP1 and negative or positive for CD8 TIL in tumor tissues of patients with MPM. *HR* hazard ratio, *CI* 98% confidence interval, *PE* pleural effusion. *p < 0.05, **p < 0.001.

Optimal cutoff values for each parameter in predicting 2-year survival determined using ROC analyses were as follows: 9.56 pg/ml for sCTLA-4, 165.73 pg/ml for sPD-L1, 601.50 pg/ml for sPD-1, 8100.75 pg/ml for TGF-β_1_, 198.28 pg/ml for TGF-β_2_, and 28.56 pg/ml for TGF-β_3_. Both TGF-β_1_ (*p* = 0.008) and TGF-β_3_ (*p* = 0.004) significantly predicted the 2-year survival in patients with MPM, whereas the other four PE parameters did not (Supplementary Fig. S5). Kaplan–Meier survival analysis demonstrated that higher TGF-β_1_ (*p* < 0.0001) and TGF-β_3_ (*p* = 0.028) levels were associated with poorer OS than were lower levels of these molecules, whereas none of the other four PE parameters and statuses of BAP1 and CD8 TIL showed any impact on the 5-year OS (Fig. 2).

### Relationship between statuses of the six PE parameters and patient characteristics in MPM

If the six PE parameters were divided as low or high levels following cutoff values for 2-year survival via the ROC curve, significant relationships were observed between statuses of sCTLA-4 and sPD-L1 (*p* < 0.001), sCTLA-4 and sPD-1 (*p* < 0.001), sCTLA-4 and TGF-β_1_ (*p* = 0.032), sPD-L1 and histology (*p* = 0.035), sPD-L1 and sPD-1 (*p* < 0.001), sPD-L1 and TGF-β_1_ (*p* = 0.010), sPD-1 and TGF-β_1_ (*p* = 0.010), TGF-β_1_ and histology (*p* < 0.001), TGF-β_1_ and TGF-β_2_ (*p* = 0.001), TGF-β_1_ and TGF-β_3_ (*p* < 0.001), and TGF-β_2_ and histology (*p* = 0.014), respectively (Supplementary Table S2).

### Univariate or multivariate analysis of the six PE parameters for 5-year OS

Cox regression analysis was performed to determine the predictive value of clinical variables for the 5-year OS. Prognostic impacts of the three soluble ICMs and three TGF-β isoforms in PE of MPM were assessed independently via univariate and multivariate analyses, with the ICMs and isoforms as confounding factors. The parameter number was set to six, as the multivariate analysis for soluble ICM and TGF-β cohorts included 72 cases with 61 events and 70 cases with 60 events, respectively. The other five parameters were sex^27^, histological subtype^27,28^, and TNM Stage^25^, which were broadly recognized as prognostic factors for MPM, including BAP1 and CD8 TIL statuses. In comparison, both BAP1 and CD8 TIL statuses were undecidable in seven cases, as biopsy was performed outside our hospital, and the TGF-β_2_ or TGF-β_3_ levels in PE were undetected in the other two cases. Univariate analysis of the soluble ICM cohort showed that histological subtype, cStage, and BAP1 status were significantly associated with 5-year OS, and multivariate analysis showed that histology and cStage were independent poor prognostic factors for 5-year OS in soluble ICM cohorts, whereas none of the soluble ICMs had any impact on 5-year OS (Supplemental Table S3). Conversely, univariate analysis for the TGF-β cohort showed histological subtype, cStage, BAP1 status, TGF-β_1_, and TGF-β_3_ to be significantly associated with 5-year OS, and multivariate analysis showed that histology and cStage were independent poor prognostic factors for 5-year OS in TGF-β cohort; both TGF-β_1_ and TGF-β_3_ were also significantly associated with 5-year OS, while TGF-β_2_ did not affect OS (Table 2).

**Table 2:** Cox proportional hazard model for OS in TGFβ cohort (n = 70).

	Univariate		Multivariate	
	Hazard ratio (95% CI)	*p* value	Hazard ratio (95% CI)	*p* value
Sex (male vs female)	0.513 (0.213–1.237)	0.137	0.660 (0.247–1.762)	0.407
Histology (epi. vs non-epi.)	0.371 (0.210–0.653)	0.001*	0.541 (0.276–1.060)	0.074
cStage (I-II. vsIII-IV and rec.)	0.381 (0.190–0.764)	0.007*	0.347 (0.157–0.766)	0.009*
BAP1 (loss vs retained)	1.792 (1.007–3.190)	0.047*	1.456 (0.779–2.719)	0.239
CD8 TIL (neg. vs pos.)	1.218 (0.720–2.063)	0.462	1.087 (0.614–1.924)	0.774
TGFβ_1_ (low vs high)	0.308 (0.169–0.560)	0.0001**	0.415 (0.215–0.802)	0.009*

	Univariate		Multivariate	
	Hazard ratio (95% CI)	*p* value	Hazard ratio (95% CI)	*p* value
Sex (male vs female)	0.513 (0.213–1.237)	0.137	0.709 (0.270–1.861)	0.484
Histology (epi. vs non-epi.)	0.371 (0.210–0.653)	0.001*	0.387 (0.206–0.736)	0.003*
cStage (I-II. vsIII-IV and rec.)	0.381 (0.190–0.764)	0.007*	0.309 (0.139–0.690)	0.004*
BAP1 (loss vs retained)	1.792 (1.007–3.190)	0.047*	1.584 (0.836–2.999)	0.158
CD8 TIL (neg. vs pos.)	1.218 (0.720–2.063)	0.462	1.188 (0.672–2.099)	0.554
TGFβ_2_ (low vs high)	0.940 (0.554–1.595)	0.819	1.023 (0.576–1.816)	0.939

	Univariate		Multivariate	
	Hazard ratio (95% CI)	*p* value	Hazard ratio (95% CI)	*p* value
Sex (male vs female)	0.513 (0.213–1.237)	0.137	0.560 (0.214–1.464)	0.237
Histology (epi. vs non-epi.)	0.371 (0.210–0.653)	0.001*	0.437 (0.233–0.821)	0.010*
cStage (I–II. Vs III–IV and rec.)	0.381 (0.190–0.764)	0.007*	0.316 (0.143–0.695)	0.004*
BAP1 (loss vs retained)	1.792 (1.007–3.190)	0.047*	2.026 (1.052–3.904)	0.035*
CD8 TIL (neg. vs pos.)	1.218 (0.720–2.063)	0.462	1.178 (0.674–2.060)	0.565
TGFβ_3_ (low vs high)	0.466 (0.272–0.801)	0.006*	0.405 (0.227–0.723)	0.002*

*OS* overall survival, *ICM* immune checkpoint molecule, *CI* confidence interval, *epi.* epithelioid, *non-epi.* non-epithelioid, *rec.* reccurence after surgery, *neg.* negative, *pos.* positive.

*p < 0.05; **p < 0.001.

### Relationships between statuses of six PE parameters and ICI efficacy in patients with MPM

We determined the predictive impact of these six parameters on ICI efficacy in patients with MPM. In our cohort, seven patients received anti-PD-1 monotherapy, two patients were administered a combination of anti-PD-1 and -CTLA4 therapies, and one patient was treated with a combination of anti-PD-1 therapy and cytotoxic chemotherapy for MPM. The six PE parameters were divided into low or high levels using the ROC curve following the cutoff values for response to anti-PD-1 monotherapy: responder (partial response or stable disease for > 6 months) or non-responder (progressive disease or stable disease for < 6 months) as per modified RECIST criteria^29^ (Supplementary Fig. S6). We found that higher sPD-1 in PE was significantly associated with responders in patients treated with anti-PD-1 monotherapy via chi-squared test, although no significant relationship was observed between the other five PE parameters and response to anti-PD-1 monotherapy or all six PE parameters and response to patients treated with ICI (Table 3).

**Table 3:** Relationship between efficacy of ICI and statuses of BAP1, CD8 TIL, and the 6 PE parameters.

		Anti-PD1 monotherapy (n = 7)			ICI (all cases, n = 10)		
		PR-long SD	PD	*p* value	PR-long SD	PD	*p* value
BAP1	Loss	4	3	unanalyzable	5	4	0.389
	Retained	0	0		1	0	
CD8 TIL	Negative	1	2	0.270	1	2	0.260
	Positive	3	1		5	2	
sCTLA4	Low	2	2	0.659	3	2	1.000
	High	2	1		3	2	
sPD-L1	Low	2	2	0.659	3	2	1.000
	High	2	1		3	2	
sPD-1	Low	1	3	0.047*	2	3	0.197
	High	3	0		4	1	
TGF-β_1_	Low	3	1	0.270	3	1	0.429
	High	1	2		3	3	
TGF-β_2_	Low	3	1	0.270	3	2	1.000
	High	1	2		3	2	
TGF-β_3_	Low	2	2	0.659	2	2	0.598
	High	2	1		4	2	

*ICI* immuno checkpoint inhibitor, *PR* partial response, *SD* stable disease, *PD* progressive disease.

*p < 0.05.

## Discussion

PE, which could reflect the TIME of MPM, is easy to collect under local anesthesia. The most popular diagnostic marker for PE in MPM is hyaluronic acid; however, its accuracy is insufficient to provide a definitive diagnosis. To assess diagnostic markers of MPM in PE, we simultaneously assessed three ICMs and three TGF-β isoforms in PE from FP and MPM. We found that PE in MPM had higher TGF-β_2_, suggesting it is a diagnostic biomarker to distinguish between FP and MPM.

A detailed understanding of the TIME in PE of MPM is important. Positive correlations were observed between levels of sCTLA-4 and sPD-L1, sCTLA-4 and sPD-1, and sPD-L1 and sPD-1 in PE of MPM. The Rs was particularly high for the relationship between sCTLA4 and sPD-1. Both CTLA-4 and PD-1 are expressed in exhausted T cells, suggesting that soluble ICMs originate from tumor-infiltrating exhausted T cells. These findings suggest that not only single ICM but multiplex ICMs help tumors to escape host immunity, which is a potential reason for the limited efficacy of ICI therapy in patients with MPM. We assessed only three soluble ICMs; however, the three soluble ICM levels positively correlated with each other, suggesting unexamined ICMs may also be rich in the PE of MPM. If CD8 + TILs are exhausted with higher multiplex soluble forms of ICM in the PE of MPM, ICI monotherapy-mediated activation of antitumor immunity may be difficult with high probability. We found that sCTLA-4 was higher in cases with CD8 + TIL than in those without CD8 + TIL, supporting the hypothesis that CD8 + TILs in the TIME of MPM were exhausted.

We assessed the relationship between clinicopathological features and six parameters in the PE of patients with MPM. We found that BAP1 status was significantly associated with TGF-β_1_ levels in PE of MPM and was associated with sPD-L1 via *t*-test, although not significantly. BAP1 is not only a tumor suppressor gene^30^ but also a regulator of proteins involved in DNA damage repair, cell differentiation, cell proliferation, and immune response^31,32^. BAP1 loss reflects BAP1 mutation^33^. Proteomic data from malignant peritoneal mesothelioma showed BAP1-deficient tumors characterized by an inflammatory TIME and immune checkpoint activation^26^. In PE of MPM with BAP1 loss, sPD-L1 was slightly lower than that in PE of MPM with BAP1 expression, suggesting that BAP1 function loss because of gene mutation directly decreased sPD-L1 secreting or indirectly inhibited membrane binding PD-L1 shedding in PE. TGF-β_1_ levels in PE were significantly lower in MPM with BAP1 loss than in MPM with BAP1 expression. This indicates that BAP1 function loss inhibits TGF-β_1_ secretion in PE, thus implying that BAP1 alterations affect the EMT and tumor immunoescape^34^, although its clinical significance remains unknown. Another potential reason for the observed significant difference in TGF-β_1_ levels between cases with BAP1 loss and retention might be that it is independent of BAP1 function but rather reflects variations in histological subtypes. Notably, BAP1 loss is strongly associated with the epithelioid subtype of MPM, which is significantly correlated with lower TGF-β_1_ levels. We also found that TGF-β_1_ in PE was higher in cases without CD8 TIL than in those with CD8 TIL. TGF-β_1_ is an immune suppressor that causes CD8 T cell exclusion from tumors in TIME^35^. The effects of BAP1 loss on EMT and tumor immune escape should be further investigated.

The potential of the six PE parameters in predicting MPM prognosis is worth considering. Although none of the three ICMs affected prognosis in patients with MPM, high TGF-β_1_ and TGF-β_3_ levels predicted poor prognoses. Our findings suggest that TGF-β may represent a promising target for MPM treatment, as it appears to fulfill dual roles in EMT and immune suppression^19,20^, which contribute to the worsening of MPM prognoses. Functional differences among three TGF-β isoforms should be investigated. TGF-β_1_ knockout mice develop a spontaneous fatal inflammatory syndrome until 3–4 weeks of age^36^, whereas both TGF-β_2_ and TGF-β_3_ knockout mice die as fetuses. TGF-β_2_ knockout mice showed developmental defects in multiple organs including cardiovascular, respiratory, and skeletal organs^36^, whereas TGF-β_3_ knockout mice exhibited retarded growth in lungs with normal cardiovascular development^37^. Theoretically, TGF-β is a promising target for anti-cancer therapy; however, the pan-TGF-β inhibitor has not been used clinically because of cardiotoxicity in animal models^38^. We showed that the three TGF-β isoforms indicated different consequences in MPM; a selective inhibition strategy for one or two of the three TGF-β isoforms may be feasible for MPM treatment. An antisense oligonucleotide targeting TGF-β_2_ inhibits lung metastasis in a preclinical in vivo mouse model^39^, which supports this strategy. The relationship between the six PE parameters and ICI efficacy in patients with MPM was of interest. Although our findings support that a higher baseline status of sPD-1 in PE predicts the response to anti-PD-1 monotherapy in patients with MPM, these findings should be interpreted with caution because of the small sample size. Therefore, further re-evaluation is warranted after accumulating a larger number of cases.

Our study has several strengths. First, this is the first study to evaluate the multiplex soluble forms of ICM and three TGF-β isoforms in PE of MPM simultaneously. Second, to the best of our knowledge, this is the largest study to evaluate sCTLA4, sPD-L1, sPD-1, and TGF-β in PE of MPM. PE sampling can be performed under local anesthesia without hospitalization and provides rapid results compared to pleural biopsy. This method may facilitate earlier diagnosis and prognosis prediction in patients with MPM. However, this study has some limitations. First, the sample size was small compared with that in similar studies on common cancer types; however, obtaining data for many patients with MPM was difficult because of its rarity. Another limitation of our study was the assessment of only six PE molecules despite the presence of numerous soluble factors in PE. Future studies should consider comprehensive PE analysis to identify diagnostic, prognostic, and predictive markers.

In conclusion, we found positive correlations among all combinations of the three soluble ICMs. In addition, positivity for CD8 + TIL in the tumor tissue was associated with higher levels of sCTLA-4 in the PE of MPM. Although none of the three soluble forms of ICMs showed a prognostic impact in patients with MPM, the baseline sPD-1 levels in PE predicted the response to anti-PD-1 monotherapy. Conversely, TGF-β_2_ in PE is a differential diagnostic marker between FP and MPM, and TGF-β_1_ and TGF-β_3_ levels are promising prognostic biomarkers for MPM.

### Supplementary Information

Supplementary Table S1.

Supplementary Table S2.

Supplementary Table S3.

Supplementary Figure S1.

Supplementary Figure S2.

Supplementary Figure S3.

Supplementary Figure S4.

Supplementary Figure S5.

Supplementary Figure S6.

Supplementary Legends.

## Data Availability

The datasets used and/or analyzed in the current study are available from the corresponding author upon reasonable request.

## Abbreviations

BAP1: BRCA1 associated protein 1
CD8 TIL: Tumor infiltrating CD8 T cell
cStage: Clinical stage
CTLA-4: Cytotoxic T-lymphocyte-associated protein 4
ELISA: Enzyme-linked immunosorbent assay
EMT: Epithelial-mesenchymal transition
FP: Fibrinous pleuritis
ICI: Immune checkpoint inhibitor
ICM: Immune checkpoint molecule
IHC: Immunohistochemistry
MPM: Malignant pleural mesothelioma
OS: Overall survival
PD1: Programmed cell death protein 1
PD-L1: Programmed cell death protein 1 ligand-1
PE: Pleural effusion
ROC: Receiver operating characteristic
Rs: Spearman’s rank correlation coefficient
sCTLA-4: Soluble form of cytotoxic T-lymphocyte-associated protein 4
sPD-1: Soluble form of programmed cell death protein 1
sPD-L1: Soluble form of programmed cell death protein 1 ligand-1
TGF-β: Transforming growth factor-β
TIME: Tumor immune microenvironment
