# Supplementary material for: Characterizing soluble immune checkpoint molecules and TGF-β1,2,3 in pleural effusion of malignant pleural mesothelioma
Source: Sci Rep. 2024 Jul 10;14:15947. doi: 10.1038/s41598-024-66189-5 (PMC11236966; doi:10.1038/s41598-024-66189-5)
Supplement: Supplementary file 4 — Supplementary Figure S1. [file 41598_2024_66189_MOESM4_ESM.pptx]

## Slide 1
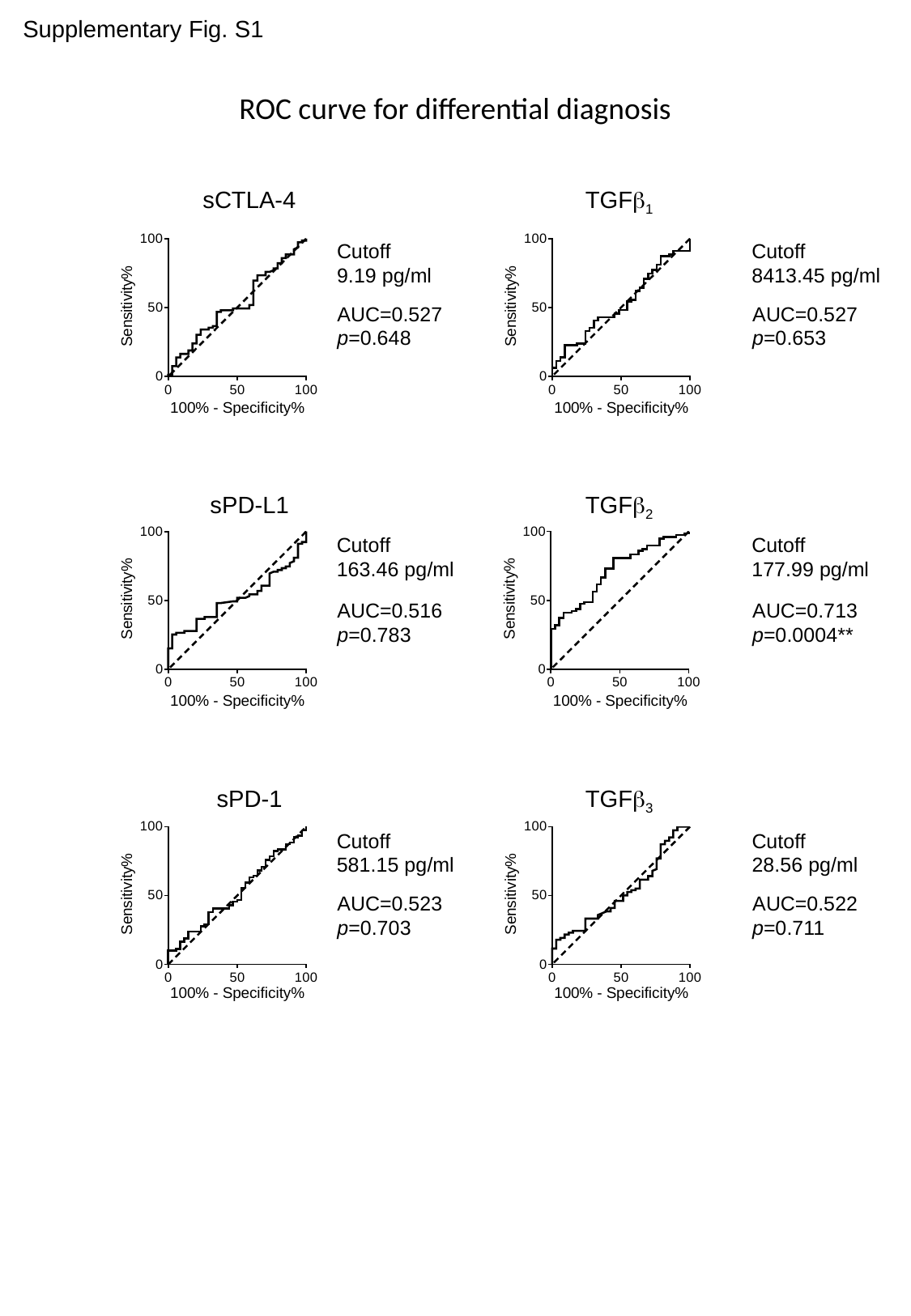

Supplementary Fig. S1
ROC curve for differential diagnosis
sCTLA-4
TGFb1
Cutoff
8413.45 pg/ml
Cutoff
9.19 pg/ml
AUC=0.527
p=0.648
AUC=0.527
p=0.653
sPD-L1
TGFb2
Cutoff
163.46 pg/ml
Cutoff
177.99 pg/ml
AUC=0.516
p=0.783
AUC=0.713
p=0.0004**
sPD-1
TGFb3
Cutoff
581.15 pg/ml
Cutoff
28.56 pg/ml
AUC=0.523
p=0.703
AUC=0.522
p=0.711
