# Supplementary figures and images for: Characterizing soluble immune checkpoint molecules and TGF-β1,2,3 in pleural effusion of malignant pleural mesothelioma
Source: Sci Rep. 2024 Jul 10;14:15947. doi: 10.1038/s41598-024-66189-5 (PMC11236966; doi:10.1038/s41598-024-66189-5)

## Slide 1
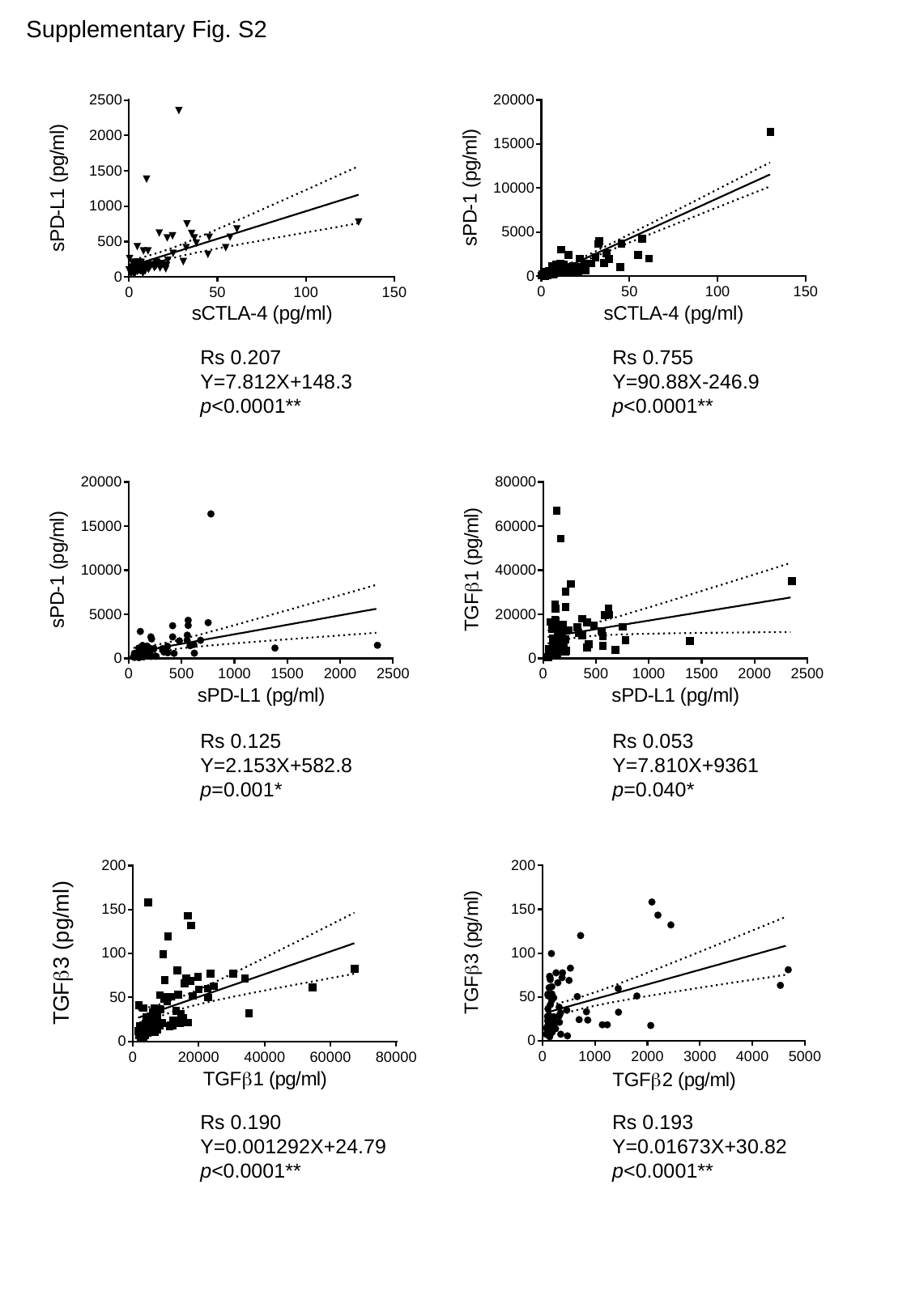

Supplementary Fig. S2
Rs 0.207
Y=7.812X+148.3
p<0.0001**
Rs 0.755
Y=90.88X-246.9
p<0.0001**
Rs 0.125
Y=2.153X+582.8
p=0.001*
Rs 0.053
Y=7.810X+9361
p=0.040*
Rs 0.190
Y=0.001292X+24.79
p<0.0001**
Rs 0.193
Y=0.01673X+30.82
p<0.0001**

Supplement: Supplementary file 5 — Supplementary Figure S2. [file 41598_2024_66189_MOESM5_ESM.pptx]

## Slide 1
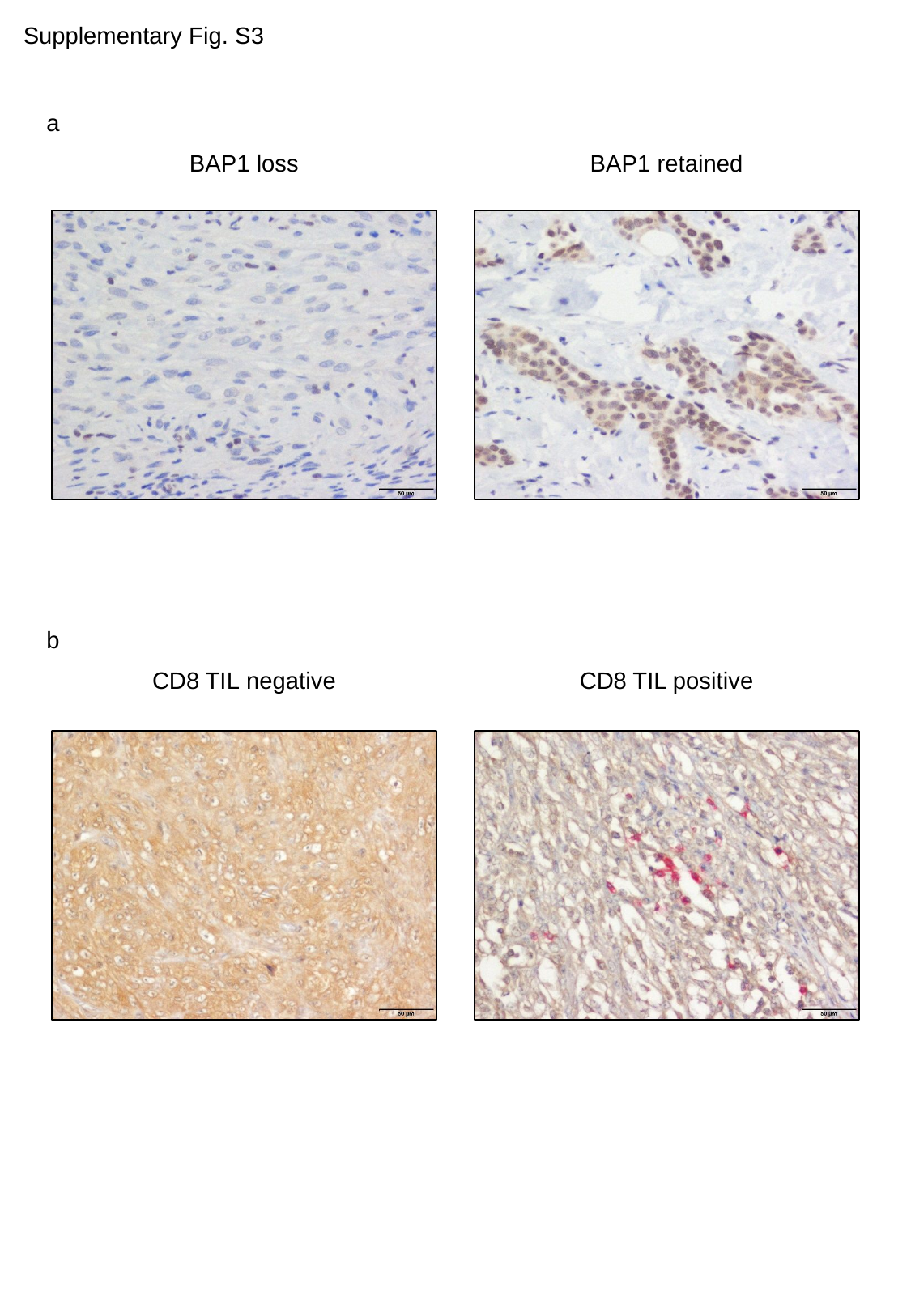

Supplementary Fig. S3
a
BAP1 loss
BAP1 retained
b
CD8 TIL negative
CD8 TIL positive

Supplement: Supplementary file 6 — Supplementary Figure S3. [file 41598_2024_66189_MOESM6_ESM.pptx]

## Slide 1
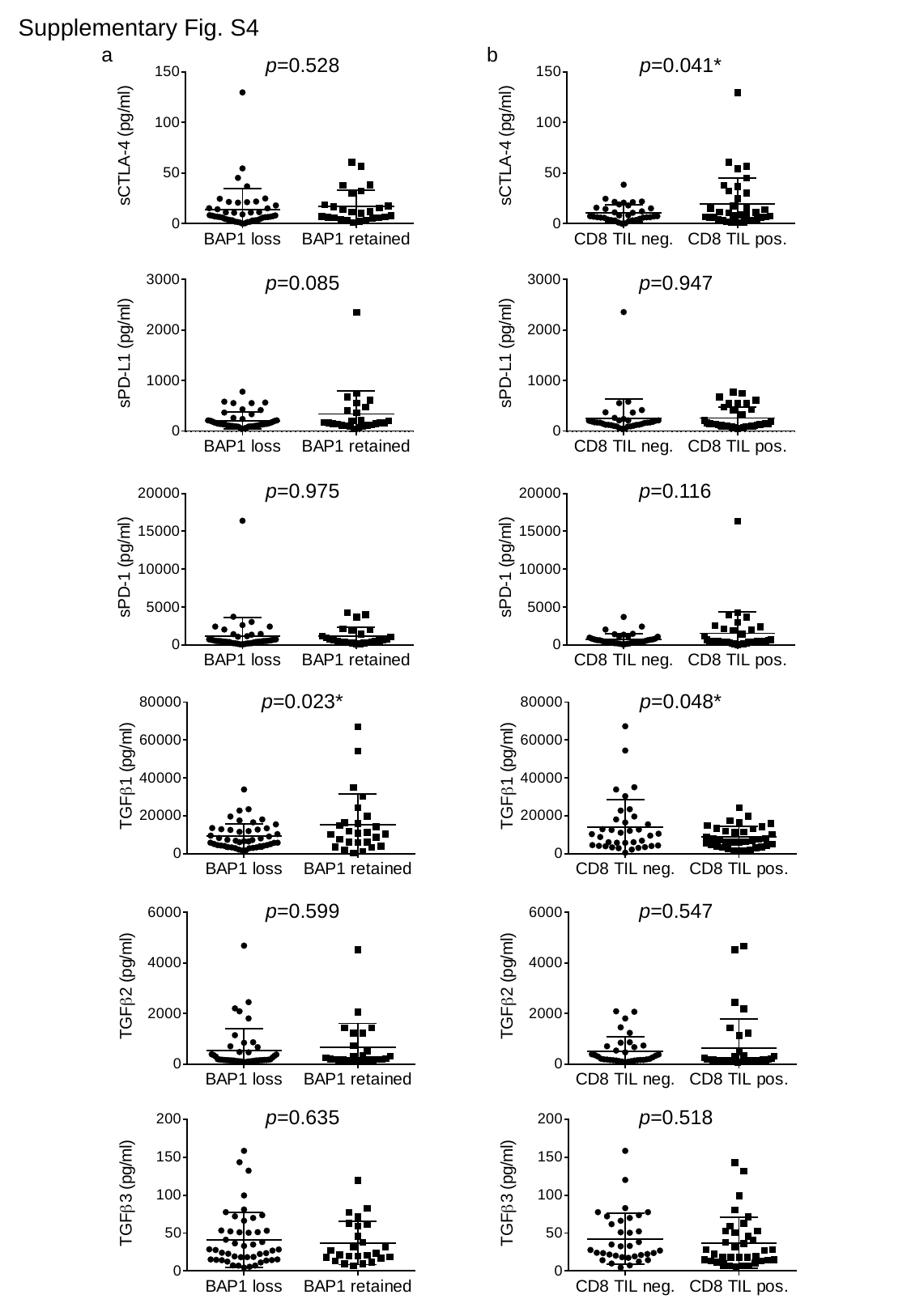

Supplementary Fig. S4
a
b
p=0.528
p=0.041*
p=0.085
p=0.947
p=0.975
p=0.116
p=0.023*
p=0.048*
p=0.599
p=0.547
p=0.635
p=0.518

Supplement: Supplementary file 7 — Supplementary Figure S4. [file 41598_2024_66189_MOESM7_ESM.pptx]
