# Supplementary material for: Characterizing soluble immune checkpoint molecules and TGF-β1,2,3 in pleural effusion of malignant pleural mesothelioma
Source: Sci Rep. 2024 Jul 10;14:15947. doi: 10.1038/s41598-024-66189-5 (PMC11236966; doi:10.1038/s41598-024-66189-5)
Supplement: Supplementary file 8 — Supplementary Figure S5. [file 41598_2024_66189_MOESM8_ESM.pptx]

## Slide 1
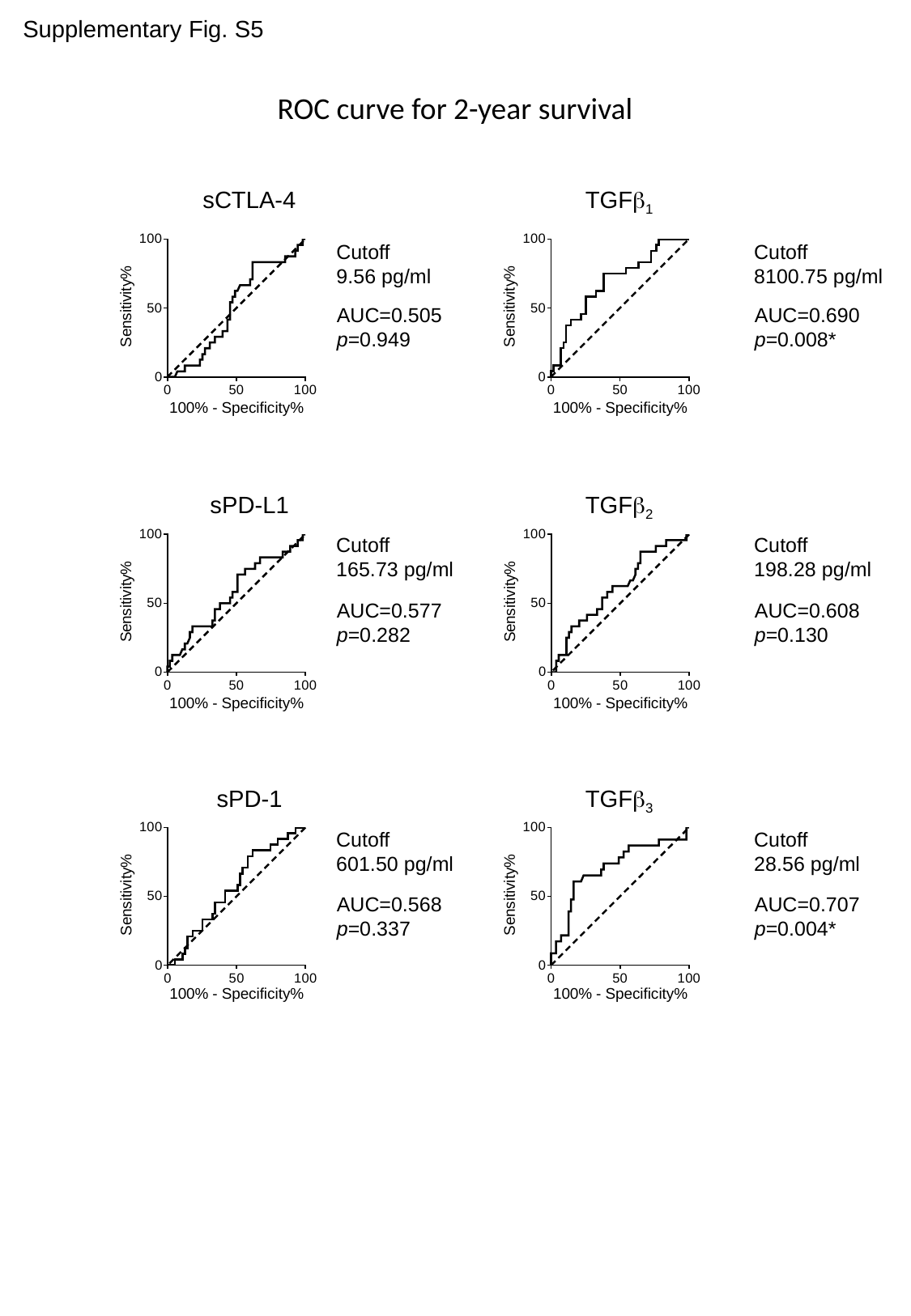

Supplementary Fig. S5
ROC curve for 2-year survival
sCTLA-4
TGFb1
Cutoff
9.56 pg/ml
Cutoff
8100.75 pg/ml
AUC=0.505
p=0.949
AUC=0.690
p=0.008*
sPD-L1
TGFb2
Cutoff
165.73 pg/ml
Cutoff
198.28 pg/ml
AUC=0.577
p=0.282
AUC=0.608
p=0.130
sPD-1
TGFb3
Cutoff
601.50 pg/ml
Cutoff
28.56 pg/ml
AUC=0.568
p=0.337
AUC=0.707
p=0.004*
