# Supplementary material for: Characterizing soluble immune checkpoint molecules and TGF-β1,2,3 in pleural effusion of malignant pleural mesothelioma
Source: Sci Rep. 2024 Jul 10;14:15947. doi: 10.1038/s41598-024-66189-5 (PMC11236966; doi:10.1038/s41598-024-66189-5)
Supplement: Supplementary file 9 — Supplementary Figure S6. [file 41598_2024_66189_MOESM9_ESM.pptx]

## Slide 1
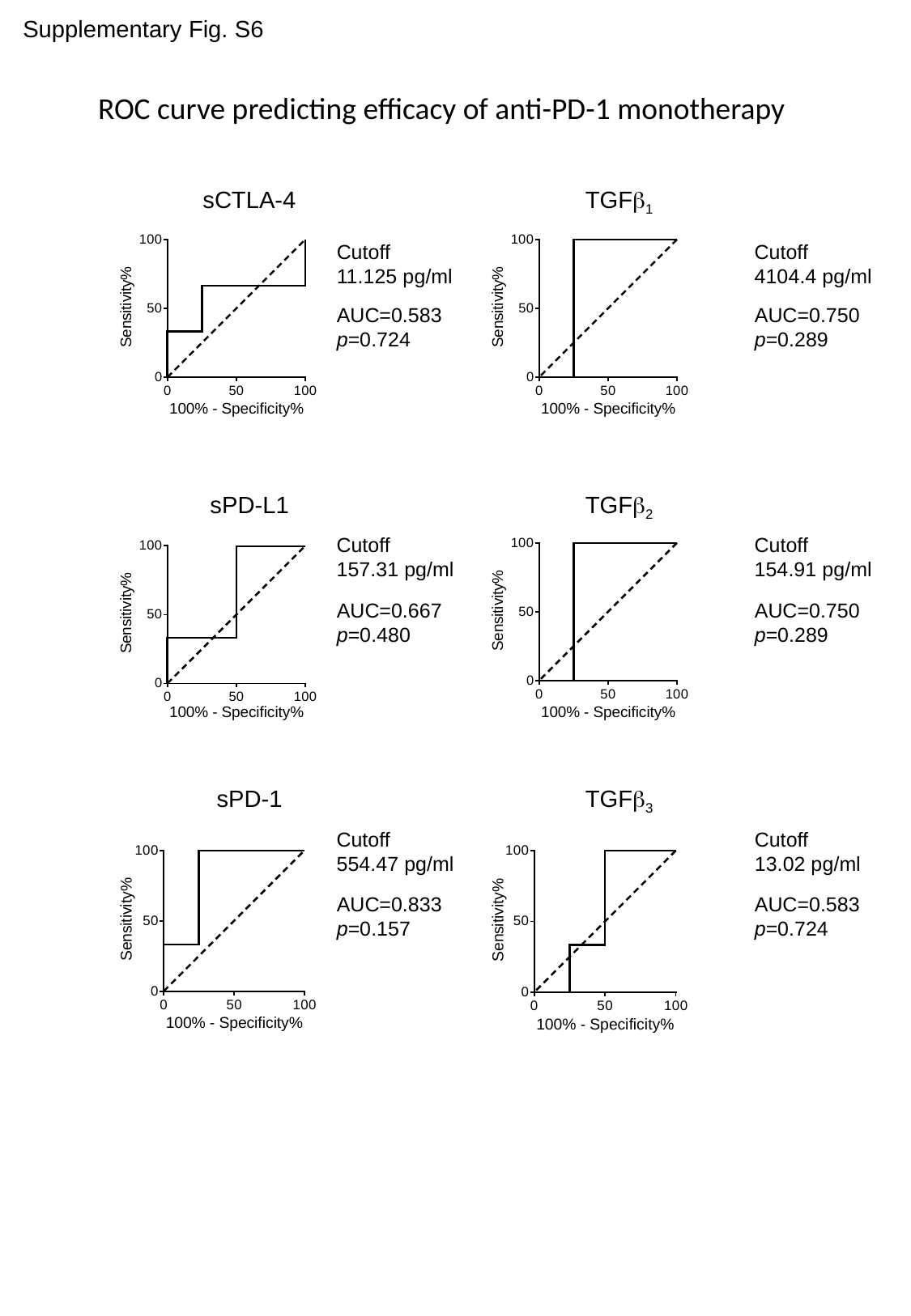

Supplementary Fig. S6
ROC curve predicting efficacy of anti-PD-1 monotherapy
sCTLA-4
TGFb1
Cutoff
11.125 pg/ml
Cutoff
4104.4 pg/ml
AUC=0.583
p=0.724
AUC=0.750
p=0.289
sPD-L1
TGFb2
Cutoff
157.31 pg/ml
Cutoff
154.91 pg/ml
AUC=0.667
p=0.480
AUC=0.750
p=0.289
sPD-1
TGFb3
Cutoff
554.47 pg/ml
Cutoff
13.02 pg/ml
AUC=0.833
p=0.157
AUC=0.583
p=0.724
