# Supplementary material for: Characterizing soluble immune checkpoint molecules and TGF-β1,2,3 in pleural effusion of malignant pleural mesothelioma
Source: Sci Rep. 2024 Jul 10;14:15947. doi: 10.1038/s41598-024-66189-5 (PMC11236966; doi:10.1038/s41598-024-66189-5)
Supplement: Supplementary file 10 — Supplementary Legends. [file 41598_2024_66189_MOESM10_ESM.docx]

**Supplementary Figure Legends**

**Supplementary Fig. S1. ROC curves for predicting differential diagnosis between FP and MPM**

ROC curves predicting the differential diagnosis between FP and MPM via predicting sCTLA-4, sPD-L1, sPD-1, TGF-β_1_, TGF-β_2_, and TGF-β_3_ are shown. ***p*<0.001. PE, pleural effusion; FP, fibrinous pleuritis; MPM, malignant pleural mesothelioma; ROC, receiver operating characteristic; AUC, area under the curve.

**Supplementary Fig. S2. Correlations between six parameters in PE of MPM**

The level of sCTLA-4 is positively correlated with sPD-L1 and sPD-1, sPD-L1 level positively correlates with sPD-1 and TGF-β_1_, TGF-β_1_ positively correlates with TGF-β_3_, and TGF-β_2_ positively correlates with TGF-β_3_. Data were analyzed using Spearman’s rank correlation test. **p*<0.05, **p<0.001. Rs: Spearman rank correlation coefficient; PE, pleural effusion; MPM, malignant pleural mesothelioma.

**Supplementary Fig. S3. IHC findings of BAP1 and CD8 TIL in MPM tissue**

Representative IHC findings of (a) BAP1 loss or retained MPM are shown (×200). (b) Representative IHC findings of double staining for CD8 TIL (red) and EGFR (brown) in for CD8 TIL negative or positive MPM are shown (×200). Bars; 50 μm. IHC; immunohistochemical reaction, BAP1; BRCA1 associated protein 1, CD8 TIL; tumor-infiltrating CD8 T cell; MPM, malignant pleural mesothelioma.

**Supplementary Fig. S4.** **Relationships between the six parameters in PE and BAP1 or CD8 TIL statuses in patients with MPM**

(a) Each dot represents levels of sCTLA4, sPD-L1, sPD-1, TGF-β_1_, TGF-β_2_, and TGF-β_3_ in PE of BAP1 loss or retained MPM. (b) Each dot represents levels of sCTLA4, sPD-L1, sPD-1, TGF-β_1_, TGF-β_2_, and TGF-β_3_ in PE of CD8 TIL negative or positive MPM. Data are analyzed using *t*-tests. Bars: Mean ± SD. **p*<0.05. PE, pleural effusion; MPM, malignant pleural mesothelioma; BAP1; BRCA1 associated protein 1, CD8 TIL; tumor-infiltrating CD8 T cell.

**Supplementary Fig. S5. ROC curves for predicting 2-year survival in patients with MPM.**

ROC curves predicting 2-year survival for sCTLA-4, sPD-L1, sPD-1, TGF-β_1_, TGF-β_2_, and TGF-β_3_ are shown. **p*<0.05. ROC, receiver operating characteristic; AUC, area under the curve; MPM, malignant pleural mesothelioma.

**Supplementary Fig. S6. ROC curves for predicting response to anti-PD-1 monotherapy in patients with MPM.**

ROC curves predicting responses to anti-PD-1 monotherapy for sCTLA-4, sPD-L1, sPD-1, TGF-β_1_, TGF-β_2_, and TGF-β_3_ are shown. ROC, receiver operating characteristic; AUC, area under the curve; MPM, malignant pleural mesothelioma.
